# Supplementary material for: Resolving the full spectrum of human genome variation using Linked-Reads
Source: Genome Res. 2019 Apr;29(4):635–45. doi: 10.1101/gr.234443.118 (PMC6442396; doi:10.1101/gr.234443.118)
Supplement: Supplemental Material [file supp_gr.234443.118_Supplemental_longranger-2.2.zip › longranger-2.2.tar.bz2!/lr2.2-public/lib/stan/cmdstan/stan/doc/api/html/agrad_2rev_2functions_2_phi__approx_8hpp_source.html]

Stan: src/stan/agrad/rev/functions/Phi\_approx.hpp Source File


|  |  |
| --- | --- |
|  | Stan  2.5.0  probability, sampling & optimization |


- Main Page
- Related Pages
- Namespaces
- Classes
- Files

- File List
- File Members

All Classes Namespaces Files Functions Variables Typedefs Enumerations Enumerator Friends Macros Pages

- src
- stan
- agrad
- rev
- functions

Phi\_approx.hpp

Go to the documentation of this file.

1 #ifndef STAN\_\_AGRAD\_\_REV\_\_FUNCTIONS\_\_PHI\_APPROX\_HPP

2 #define STAN\_\_AGRAD\_\_REV\_\_FUNCTIONS\_\_PHI\_APPROX\_HPP

3

4 #include <stan/agrad/rev/var.hpp>

5 #include <stan/agrad/rev/internal/precomp\_v\_vari.hpp>

6 #include <stan/math/functions/inv\_logit.hpp>

7

8 namespace stan {

9  namespace agrad {

10

48  inline var Phi\_approx(const stan::agrad::var& a) {

49  // return inv\_logit(0.07056 \* pow(a,3.0) + 1.5976 \* a);

50

51  double av = a.vi\_->val\_;

52  double av\_squared = av \* av;

53  double av\_cubed = av \* av\_squared;

54  double f = stan::math::inv\_logit(0.07056 \* av\_cubed + 1.5976 \* av);

55  double da = f \* (1 - f) \* (3.0 \* 0.07056 \* av\_squared + 1.5976);

56  return var(new precomp\_v\_vari(f,a.vi\_,da));

57  }

58

59  }

60 }

61 #endif

precomp\_v\_vari.hpp

stan::agrad::Phi\_approx

var Phi\_approx(const stan::agrad::var &a)

Approximation of the unit normal CDF for variables (stan).

**Definition:** Phi\_approx.hpp:48

stan::agrad::vari::val\_

const double val\_

The value of this variable.

**Definition:** vari.hpp:37

stan::math::inv\_logit

boost::math::tools::promote\_args< T >::type inv\_logit(const T a)

Returns the inverse logit function applied to the argument.

**Definition:** inv\_logit.hpp:52

stan::agrad::var::vi\_

vari \* vi\_

Pointer to the implementation of this variable.

**Definition:** var.hpp:40

var.hpp

stan::agrad::precomp\_v\_vari

**Definition:** precomp\_v\_vari.hpp:11

stan::agrad::var

Independent (input) and dependent (output) variables for gradients.

**Definition:** var.hpp:27

inv\_logit.hpp


---

[ Stan Home Page ]

*© 2011–2014,
Stan Development Team.*
